# High-quality genome assembly of *Listeria monocytogenes* Lm208 (CNL895805): a domestic strain able to grow faster at low temperature when exogenous unsaturated fatty acids are present

**DOI:** 10.1128/mra.00816-24

**Published:** 2025-07-09

**Authors:** Maud Darsonval, Hélène Gardon, Aurore Quilleré, Philippe Velge, Laurent Guillier, Florence Dubois-Brissonnet

**Affiliations:** 1 Université Paris-Saclay, INRAE, AgroParisTech, MICALIS Institute, Jouy-en-Josas, France; 2 Université Paris-Saclay, INRAE, AgroParisTech, UMR SAYFOOD 27048, Palaiseau, France; 3 INRAE, Université de Tours, ISP 27092, Nouzilly, France; 4 Direction de l’évaluation des risques, Agence nationale de sécurité sanitaire de l'alimentation de l'environnement et du travail (ANSES) 55036, Maisons-Alfort, France; University of California Riverside, Riverside, California, USA

**Keywords:** *Listeria monocytogenes*, fatty-acid metabolism, genome analysis

## Abstract

The genome of *Listeria monocytogenes* Lm208 strain has been sequenced and assembled. The draft genome has been automatically annotated. Considering its specific ability to grow faster at low temperatures in the presence of exogenous fatty acids, the annotation of genes related to fatty-acid metabolism was investigated using local and multiple sequence alignments.

## ANNOUNCEMENT

*Listeria monocytogenes* is a foodborne pathogen responsible for listeriosis. Lmo208 (CNL895805, serotype 1/2a), isolated in 1992 from a sheep’s brain, can grow faster at low temperature in the presence of exogenous unsaturated fatty acids (FA) (1, 2). Genomic DNA was extracted and purified from a 16-h culture in Tryptic Soy Broth (TSB) using the Wizard Genomic DNA Purification Kit (Promega, France). DNA sequencing was performed at the “Institut du Cerveau et de la Moëlle” (Paris, France). After quantification, DNA-seq libraries were prepared with the Nextera XT DNA Flex Library kit (Illumina, San Diego, USA). Whole Genome Sequencing (WGS) was performed on the NextSeq 500 system using a paired-end read length of 2 × 150 bp with the Illumina High Output kits. Raw reads (1,217,365 sequences; 151 bp) were analyzed using tools of the Galaxy portal (https://galaxy.migale.inrae.fr/). Trimming was performed with the following parameters: Initial IlluminaClip, Leading 3, Trailing 3, Sliding Window 4–20, and Minlen 125 bp (Trimmomatic v39.0) (3). The quality of 421,351 trimmed reads was checked with fastQC (v0.11.9) (4). *De novo* assembly was performed using SPAdes (v3.15.4) and followed by quality control (Quast v5.2.0) (5, 6). After ordering by progressiveMauve based on the genomic sequence of *L. monocytogenes* EGD-e (26 February 2015) (7), the size of the genome was 2,871,758 bp made of 12 contigs with a GC content of 37.9%, N50 value of 1,458,283 bp, L50 of 1, and an average coverage of 128×. Automatic annotation was performed with Prokka (v1.14.6) using the proteome of *L. monocytogenes* EGD-e as reference (UP000000817) (8).

2,824 predicted protein-coding genes were detected with 49 tRNAs and 4 rRNAs. 2,686 Coding Sequences (CDS) were assigned to a gene name based on the locus tag of *L. monocytogenes* EGD-e. Fifty CDS were identified using *Listi*Wiki, ListeriOmics, or NBCI’s database (9, 10). After enrichment, the remaining 120 CDS encoded hypothetical proteins. Lm208 does not harbour antibiotic resistance genes nor plasmids (Staramr v0.7.2.1) (11). Two phage regions were identified with PHASTEST (https://phastest.ca/): the *Listeria* phage A118 (NC_003216) prophage in the *comK* integration site and a partial *Streptococcus* phage T12 (NC_028700) interrupting the locus *lmo208_02875* (12–14). Contigs No. 3, 13, and 15 displayed repeated consensus regions of CRISPR and their associated caspase proteins (CRISPRCasFinder; https://crisprcas.i2bc.paris-saclay.fr/) (15). Clonal complex (CC) and lineage were defined *in silico* using the BIGSdb database (http://bigsdb.pasteur.fr/listeria), which revealed that Lm208 belongs to lineage II and CC7, clones rarely related to food isolates (16). Lm208 highlights high similarity with the four reference strains with a maximum of average nucleotide identity (ANI) at 99.99% with *L. monocytogenes* 10403S and a minimum of ANI at 94.94% with *L. monocytogenes* ScottA (FastANI (v1.3) (Table 1; 17, 18 ).

**TABLE 1:** Genome metrics of *L. monocytogenes* Lm208 and comparison to domestic reference strains EGD-e, EGD, ScottA, LO258, and 10403S

Strain	Genomic element^*a*^	RefSeq	ANI (%)	Contig no.	Length (Mb)	G + C content (%)	No. of CDS	No. of tRNA	No. of rRNAs	Source	CC
Lm208	Chr	GCF_048381475.1	100	12	2.87	37.9	2,824	49	4	Sheep’s brain	7
EGD-e	Chr	GCF_000196035.1	98.94	1	2.94	38.0	2,867	67	18	Derivative of EGD	9
EGD	Chr	GCF_000582845.1	99.93	1	2.91	38.0	2,818	67	18	Guinea pig tissue	7
10403S	Chr	GCF_000168695.2	99.99	1	2.90	38.0	2,802	67	6	Human skin lesions	7
ScottA	Chr	GCF_000212455.1	94.94	5	3.03	38	2,934	67	6	Listeriosis outbreak	7
LO28	Chr	GCF_021378215.1	98.90	1	2.98	38	2,911	67	6	Pregnant carrier	9

^*a*^ Chr: chromosome.

After automatic annotation, the annotation of Fak and Fab proteins, related to FA metabolism, was deeply investigated based on literature and local or multiple sequence alignments (1). One FakA and two FakB proteins were identified by BLASTp using the well-described *Staphylococcus aureus* FakAB system (Q2FHL2.1; ABD20605.1; ABD22554.1) as query sequences (18, 19). Eleven Fab proteins were found by Prokka or by NCBI’s BLASTp database and multiple sequence alignment (ClustalOmega v1.2.4) with 114 Fab sequences from *Streptococcaceae* and *Bacillaceae* (20). Phenotypic investigation is needed to identify the genetic marker related to its specific ability to grow faster in the presence of unsaturated FA.

## Data Availability

This whole-genome shotgun project has been deposited at GenBank under the accession number (JBLUWX000000000) and in the NCBI Sequence Read Archive (SRA SRR29855365) under BioProject accession number PRJNA1074122 and BioSample number SAMN39851069, respectively. The version described in this paper is the first version (JBLUWX000000000.1).
